# Assessment of Well-Being Among Healthcare Workers and Identification of Possible Improvement Measures Using the NIOSH Well-Being Questionnaire

**DOI:** 10.1097/JOM.0000000000003641

**Published:** 2025-12-10

**Authors:** Luca Fontana, Ivan Borrelli, Guglielmo Forgeschi, Veruscka Leso, Mauro Fedele, Paolo Emilio Santoro, Ivo Iavicoli

**Affiliations:** 1 From the Department of Public Health, Section of Occupational Medicine, University of Naples “Federico II,” Naples, Italy (L.F., G.F., V.L., M.F.); 2 Dipartimento di Sicurezza e Bioetica, Catholic University of Sacred Heart, Rome, Italy (I.B., P.E.S., I.I.); 3 Fondazione Policlinico Universitario A. Gemelli, IRCCS, Rome, Italy (I.I.).

**Keywords:** health promotion, healthcare workers, mental and physical health, Total Worker Health model, work evaluation and experience, workplace support, worker well-being

## Abstract

**Objective:**

This cross-sectional study conducted an integrated assessment of well-being among healthcare workers, investigating occupational health and safety factors, living determinants, and psychophysical health status.

**Methods:**

The Worker Well-Being Questionnaire (NIOSH WellBQ) was distributed (April–May 2024) to 319 healthcare professionals engaged in surgical divisions, medical departments, rehabilitation units, and outpatient services. Questionnaires were collected, guaranteeing anonymity and privacy of participants.

**Results:**

Findings indicated that healthcare workers were generally satisfied with their jobs and felt supported by their supervisors and colleagues. Overall, participants reported relatively few days of poor mental or physical health and a very low mean score for chronic health conditions. Finally, the results suggest that this working population is also pleased with its overall living conditions.

**Conclusions:**

The NIOSH WellBQ is a practical tool that can be used for establishing benchmarks of healthcare workers’ well-being and identifying targeted interventions for improvement.

## Abstract 2

This study assessed the well-being of healthcare workers using the NIOSH WellBQ, an innovative tool capable of providing an integrated and holistic assessment. Findings support the usefulness of applying this tool in workplaces, especially with regard to identifying the weakest areas and issues that prevent workers and companies from thriving.

## Graphical abstract

**Figure:**
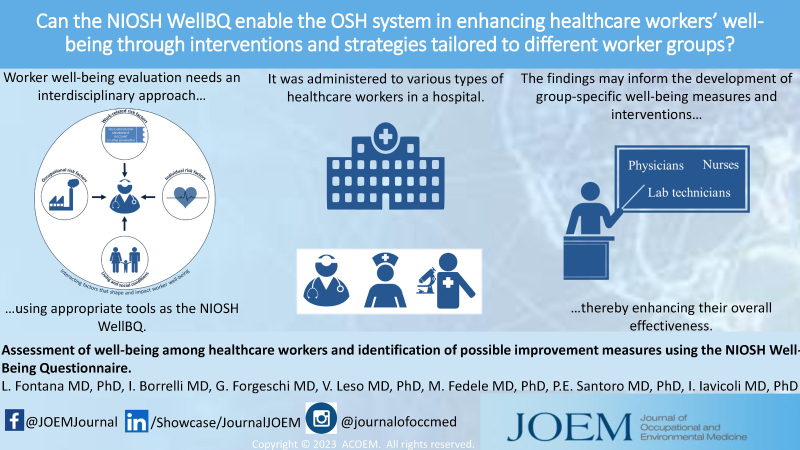

**LEARNING OUTCOMES**

After completing this enduring educational activity, the learner will be better able to:

- Define an appropriate recruitment and administration strategy for the NIOSH WellBQ;
- Analyze the specific weight and interactions of different types of determinants (occupational, work-related, individual and social) in influencing worker well-being
- Design interventions, strategies and policies to improve well-being that, being based on the needs of specific groups within the same workforce, are more effective.

In recent years, there has been a growing recognition in the occupational safety and health (OSH) field of the necessity to develop innovative and integrated models for the prevention and protection of workers’ health and safety in order to achieve continuous improvement in the health, safety, and well-being of employees in the workplaces.^1–4^ This awareness emerged and evolves from several key considerations.

Firstly, despite significant advancements in the assessment and management of classical occupational risk factors, workplace accidents and occupational diseases still remain a pervasive social issue of unacceptable magnitude, since in 2019, approximately 2.9 million workers worldwide lost their lives due to work-related causes (with 2.6 million and 300,000 deaths, respectively, due to work-related diseases and occupational injuries).^5,6^ Second, increasing scientific evidence showed that occupational risk factors might contribute to health issues and disorders previously thought to be unrelated to work, whereas, at the same time, various determinants that fall outside the conventional classification of occupational risks (such as wages, working hours, company benefits, workload, nature of the labor contract, and other employment conditions) can negatively impact workers’ health.^7,8^ Third, the recent COVID-19 pandemic has starkly underscored what many OSH professionals have long suspected, that is, a wide range of external factors (even those that cannot be narrowly defined even as work related), including pandemics, migration, informal work, globalization, and climate change that can significantly impact working conditions, leading to profound consequences, not only in terms of occupational health and safety but also in the overall well-being of workers.^2,3^ Finally, although the nature of Occupational Medicine remains fundamentally preventive, it is essential to recognize that this discipline is continuously evolving because advancements in technology, modifications in workplace environments and production processes, the emergence of new occupational hazards and diseases, and changes in health and safety regulations all contribute to its ongoing transformation.^9^

Therefore, taken together, these considerations highlight the need for an expanded role for OSH professionals and occupational physicians addressed to focus on developing preventive and protective strategies or methodologies that integrate all aspects of work into comprehensive interventions, thus ensuring a holistic approach to workers’ safety, health, and well-being. In particular, this effort should be primarily aimed at assessing and managing specific occupational hazards/risk factors whose negative impact on workers’ health and safety resulted mainly from a complex interplay and reciprocal influences between working conditions, work-related drivers, societal factors, and individual determinants, such as, for example, the psychosocial risk factors that go hand in hand with the experience of work-related stress.^10–14^ In this perspective, in an ever-changing world (which inevitably also changes the world of work), a modern and up-to-date Occupational Medicine must be able to effectively fulfill its primary mission of preventing occupational risks, but it must also be capable of addressing health and non-health issues that could hinder workers’ ability, thus providing comprehensive solutions and promoting multidisciplinary approaches to the overall well-being of the workforce.^4,15^

In this context, the *Total Worker Health®* (TWH) program created by the US National Institute for Occupational Safety and Health (NIOSH) represents a reference model at an international level that, through the definition and implementation of innovative and integrated policies, programs, and practices, aims to safeguard workers from accidents at work and occupational diseases and simultaneously to implement effective health promotion programs that improve their overall well-being.^16,17^ The cornerstone of this approach is the consideration that work is a powerful social determinant of health, but at the same time, it also recognizes the need to consider the impact that all risk factors (e.g., occupational, work-related and individual) can have on a worker’s health, particularly in view of their potential interactions and synergies.^18^ Therefore, from this point of view, applying the TWH principles extends the preventive action of Occupational Medicine. This is achieved through a global and interdisciplinary analysis of all determinants that could threaten the safety, health, and well-being of workers, and in other words, it can be considered a sort of evolution or actualization of the classic and traditional approach of Occupational Medicine based on the assessment and management occupational risk factors process.^19^

For these reasons, this study applied the TWH approach, utilizing the NIOSH Worker Well-Being Questionnaire (NIOSH WellBQ), to conduct a comprehensive assessment of well-being among healthcare workers. A prior investigation utilizing the NIOSH WellBQ across both rural and urban hospital environments revealed marked disparities in employee well-being.^20^ In rural settings, predominant concerns included limited time availability, heavy workloads, and elevated stress levels, whereas urban hospitals exhibited challenges related to occupational fatigue and diminished job engagement. Further distinctions emerged: rural institutions faced issues in employee benefits, health initiatives, and scheduling flexibility, whereas urban counterparts struggled with unsupportive workplace cultures and a lack of trust in management. Across both contexts, issues about the physical work environment and safety climate were consistently reported, encompassing experiences of sexual harassment, physical aggression, and bullying. Health-related concerns also varied: rural hospitals were associated with heightened stress and poor mental health outcomes, whereas urban facilities reported a higher prevalence of chronic conditions and hazardous alcohol consumption. Against this backdrop, the present study seeks to generate actionable insights that can guide the formulation and implementation of innovative, integrated strategies, and policies aimed at enhancing and sustaining the physical, mental, and social well-being of healthcare workers.

## METHODS

### Setting and Participants

The study was conducted in a hospital located in a large metropolitan city in Middle Italy providing a representative and adequate setting for assessing well-being in a healthcare workplace. This hospital upholds the highest standards in workplace safety, operational efficiency, and quality healthcare services because it has earned the Healthcare Accreditation of Excellence from the Joint Commission International (that requires compliance with rigorous standards, including those related to occupational health and safety, as well as staff qualifications and training), and it maintains compliance with both the UNI EN ISO 45001:2018 (occupational health and safety management systems—requirements and guidance for use) and the UNI EN ISO 9001:2015 (internationally recognized quality management system standard). Furthermore, in this regard, it is worth emphasizing that, beyond the legal obligations currently in force in Italy regarding workplace health and safety (i.e., Legislative Decree 81/08), the OSH system of this healthcare facility was already proactively involved in activities related to workers’ health promotion, training, and education. Concerning the hospital’s healthcare services, they are organized into four distinct areas of expertise, which include the surgical divisions, the medical departments, the rehabilitation units, and outpatient services. At the time of the study, the potential participant pool of healthcare professionals, including a diverse range of professional duties, consisted of 134 physicians, 157 nurses, 33 technical personnel, and 72 social and health workers (whose primary responsibilities include assisting patients with basic daily needs such as personal hygiene, dressing, eating, and mobility). Other professional jobs, such as white collars (*n* = 60) and nonphysician managers (i.e., pharmacist, biologist, or medical physicist in number of 10) were also present in the hospital.

### Recruitment Strategy and Data Collection

Aligned with the core principles of TWH and in order to maximize participant recruitment, the administration of the NIOSH WellBQ questionnaire was preceded by several informational meetings that were designed to present and discuss the survey instrument, outline the administration strategy, explain the data collection process, and clarify the subsequent methods of information processing and interpretation. These preparatory meetings, essential for the successful completion of the study, were attended by physicians with expertise both on Occupational Medicine and TWH, employer’s representative, hospital managers and supervisors, workers’ safety representatives, the head and staff of the Prevention and Protection Service, thus ensuring a shared understanding of the study objectives, methodology, and implementation process and at the same time fostering transparency, collaboration, commitment, and engagement. With regard to the recruitment strategy, considering that the research project focused on assessing the well-being of healthcare workers, the questionnaire was not proposed to white collars. Additionally, healthcare workers who worked at the hospital on an extremely occasional basis (once every 15 days or less), because of their particular contractual status (e.g., freelance contract), were excluded from the pool of potential participants because their responses to the questionnaire could have introduced significant bias compared to daily workers, who represented the target population with the most representative and reliable exposure conditions (in line with this criterion, the questionnaire was proposed to 29 freelance workers). Consequently, recruitment was actually carried out on 319 workers (distributed as follows: 130 in surgical divisions, 64 in medical departments, 62 in rehabilitation units, and 63 in outpatient services, representing 68.45% of the total workforce) compared to the 466 subjects who were employees of the hospital at the time the study was performed.

Then, the questionnaire was disseminated across all categories of healthcare workers within the hospital’s various work settings (surgical, medical, rehabilitation, and outpatient services areas). The administration and collection of the questionnaires was conducted from April to the end of May 2024. Before the administration of the questionnaire, adequately trained and instructed medical personnel provided the subjects who agreed to participate in the study with explanations regarding the correct fulfillment of the NIOSH WellBQ, its meaning, and the items it contained. Therefore, after being informed about the aims of the study and the related data processing procedures, subjects declared their written informed consent to both participation in the study and possible future publication of the data in an anonymous and collective form. The collection of completed questionnaires was carried out guaranteeing anonymity. In this regard, to ensure participants’ privacy and anonymity, a structured collection procedure was implemented, and it consisted in designating specific collection points in the aforementioned work areas (surgical divisions, medical departments, rehabilitation units, and outpatient services), each featuring two separate boxes, one for informed consent forms and another for completed questionnaires. This collection methodology enabled the secure and confidential submission of questionnaire data, which could then be organized and analyzed by groups of workers belonging to different hospital departments. The protocol study was approved by the Ethics Committee of the University Federico II–AORN Cardarelli, Naples, Italy (protocol 16082/2022), and the “Strengthening the Reporting of Observational Studies in Epidemiology” (STROBE) guideline for cross-sectional studies was used to prepare this study. The dataset and its preliminary data were used for a second-level university master’s degree thesis.^21^

### The NIOSH WellBQ

In this study, we used the validated Italian-language version of the NIOSH WellBQ.^22^ This questionnaire was designed and validated by NIOSH, and currently, it is considered the most thorough tool for assessing and measuring worker well-being.^23–25^ Administering the NIOSH WellBQ by homogeneous groups and comparing the results makes it possible to design tailored interventions, thus maximizing their effectiveness.

According to the NIOSH guidelines, a scoring system was applied to each question. The scoring scheme predominantly follows a pattern where higher scores correspond to positive outcomes related to well-being, whereas lower scores indicate negative conditions. However, some questions are reverse-coded, meaning that higher scores indicate more adverse conditions, inverting the typical scoring pattern (for example, in question 13, which investigates the fatigue perceived by workers, a high score, such as 7, indicates that the respondent experiences fatigue at work every day). For these reverse-coded items, scoring was adjusted by assigning a negative value, effectively multiplying the original score by −1. This transformation ensures that all questions are oriented such that higher scores consistently indicate better well-being, whereas lower scores reflect poorer conditions. For multi-item questions or subdomains comprising multiple items, the mean value was calculated and subsequently rounded up to the nearest whole number.

### Statistical Analysis

Statistical analysis was carried out by considering only variables coded as numerical. Sociodemographic characteristics of the participants were summarized using frequencies and percentages and stratified by hospital area of expertise (surgical divisions, medical departments, rehabilitation units, and outpatient services). Descriptive statistics, including mean, standard deviation (SD), and range, were calculated for all continuous variables to summarize the distribution of responses. Differences in item mean scores across the four hospital areas were assessed using analysis of variance (ANOVA). The level of statistical significance was set at *P* < 0.05. All statistical analyses were performed using Stata version 16.0 (StataCorp, College Station, TX).

## RESULTS

### Sociodemographic Characteristics of Participants

Of the 319 questionnaires distributed to the various hospital departments, 200 were completed and returned, resulting in a total response rate of 62.7% (53.8% in surgical divisions, 78.1% in medical departments, 71.0% in rehabilitation units, and 57.1% in outpatient services). In Table 1, we reported the main sociodemographic characteristics of healthcare workers recruited in the study. In this regard, it should be noted that the possibility of capturing this type of data (e.g., type of work, length of service, age, education level, ethnicity, gender, household income, and marital status) lies essentially in filling out the last section of the NIOSH WellBQ (i.e., optional items) that investigates the current working arrangements, occupation, industrial sector, and several basic demographic information of the respondent. Unfortunately, these questions are placed at the end of the questionnaire (after item 68), and given the length of the questionnaire and the time it takes to complete it (at least 15–20 minutes), it is not uncommon for subjects to leave this section largely incomplete. Indeed, in our study, the vast majority of participants did not answer the optional questions. Therefore, the limited sociodemographic data shown in the table were obtained from informed consents and cross-referenced with information available from the hospital administration. These data were processed collectively and, thanks to how the questionnaires and informed consents were returned (two separate boxes, one for informed consent forms and another for completed questionnaires), the privacy of workers filling out the NIOSH WellBQ was respected, as it would have been impossible to associate an individual informed consent form with the respective completed questionnaire.

**TABLE 1.** Available Sociodemographic Features of Healthcare Workers Recruited in the Study Subdivided According to their Allocation to Different Hospital Areas of Expertise

Variables	Surgical Divisions	Medical Departments	Rehabilitation Units	Outpatient Services	Total*	*P* (<0.05)
Gender						
Male, *n* (%)	14 (28)	14 (28)	13 (26)	9 (18)	50 (100)	n.s.
Female, *n* (%)	51 (36.7)	33 (23.7)	30 (21.6)	25 (18)	139 (100)	
Age (yr)						
20–29, *n* (%)	16 (33.4)	17 (35.4)	9 (18.7)	6 (12.5)	48 (100)	**0.017**
30–39, *n* (%)	16 (38.1)	12 (28.6)	10 (23.8)	4 (9.5)	42 (100)	
40–49, *n* (%)	13 (39.4)	7 (21.2)	8 (24.2)	5 (15.2)	33 (100)	
50–59, *n* (%)	12 (27.9)	7 (16.3)	10 (23.2)	14 (32.6)	43 (100)	
>59, *n* (%)	8 (34.8)	4 (17.4)	6 (26.1)	5 (21.7)	23 (100)	
Job task						
Physicians, *n* (%)	12 (44.5)	6 (22.2)	3 (11.1)	6 (22.2)	27 (100)	**<0.001**
Nurses, *n* (%)	38 (37.6)	27 (26.7)	22 (21.8)	14 (13.9)	101 (100)	
Technical personnel, *n* (%)	—	2 (9.5)	8 (38.1)	11 (52.4)	21 (100)	
Social and health workers, *n* (%)	15 (37.5)	12 (30)	10 (25)	3 (7.5)	40 (100)	
Length of service (yr)						
≤5, *n* (%)	36 (35.3)	36 (35.3)	21 (20.6)	9 (8.8)	102 (100)	**<0.001**
6–15, *n* (%)	21 (58.4)	2 (5.5)	7 (19.4)	6 (16.7)	36 (100)	
16–25, *n* (%)	5 (15.6)	5 (15.6)	9 (28.2)	13 (40.6)	32 (100)	
≥26, *n* (%)	3 (15.8)	4 (21.0)	6 (31.6)	6 (31.6)	19 (100)	

Significant values are marked with bold type.

* The numbers and percentages refer to the total of the specific variable taken into consideration.

n.s., not significant.

Female workers represented the majority of the studied population (73.5%) that, in turn, was evenly distributed across the different age groups, although individuals over 59 years were the least represented (12.2%). With regard to job tasks, nurses accounted for 53.4%, social and health workers for 21.2%, physicians for 14.3%, and technical personnel for 11.1% of the sample. Notably, more than half of the participants (54%) reported less than (or at least) 5 years of professional experience, whereas the remaining participants had a length of service of 6–15 years (19%), 16–25 years (16.9%), or more than 26 years (10.1%). A vast majority of healthcare workers were employed with a permanent or standard dependent employment contract (only 14 respondents were freelance workers).

### Mean Scores of the Total Sample

The questionnaire was structured into several conceptual domains aimed at exploring various aspects of workers’ well-being including (i) the work evaluation and experience (16 questions), (ii) the workplace policies and culture (14 questions), (iii) the workplace physical environment and safety climate (10 questions), (iv) the health status (23 questions), and (v) home, community, and society aspects (5 questions).

Most of the mean scores observed for the items in the first domain showed that, overall, healthcare workers have a positive perception of work evaluation and experience (Table 2, Section A). More specifically, participants are satisfied with their job (2.97 ± 0.72), and this fulfillment is evident through their frequent expression of positive feelings (4.72 ± 1.44) and their strong commitment to their work activities (5.22 ± 1.10). Other positive aspects included the impression of job security (2.73 ± 0.79) and the fact that they can count on the support of their supervisors, but also colleagues (3.22 ± 0.79 and 3.46 ± 0.70, respectively). On the other hand, participants reported relatively low satisfaction with their wage, benefits, and advancement (2.18 ± 0.75, 2.25 ± 0.92, and 2.34 ± 0.87, respectively). Moreover, workers indicated fatigue associated with job tasks (−4.89 ± 1.34; almost once a week), and they expressed moderate agreement with the statement that they never have enough time to complete all their work (−3.04 ± 0.85).

**TABLE 2.** Mean Scores and Standard Deviation (SD) of Different Items Involving the Entire Healthcare Workers Recruited in the Study

Items	Mean Scores ± SD	Scoring Range	Missing (n)*	Not-Applicable Responses (n)*	Items	Mean Scores ± SD	Scoring Range	Missing (n)*	Not-Applicable Responses (n)*
Section A—Domain: Work evaluation and experience									
Job satisfaction Q1	2.97 ± 0.72	1–4	1	—	Job autonomy Q8	2.74 ± 0.83	1–4	2	—
Wage satisfaction Q2	2.18 ± 0.75	1–4	0	—	Time paucity/Work overload Q9	−3.04 ± 0.85	−(1–4)	1	—
Benefits satisfaction† Q3	2.25 ± 0.92	1–4	1	14	Meaningful work Q10–11	3.51 ± 0.50	1–4	0–0	—
Adv. satisfaction Q4	2.34 ± 0.87	1–4	3	—	Positive affect Q12A–B	4.72 ± 1.44	1–7	4–4–4–4	—
Supervisor support† Q5	3.22 ± 0.79	1–4	0	6	Negative affect Q12E–H	−3.24 ± 1.26	−(1–7)	4–4–4–4	—
Coworker support Q6	3.46 ± 0.70	1–4	0	0	Work-related fatigue Q13	−4.89 ± 1.34	−(1–7)	2	-
Job Security Q7	2.73 ± 0.79	1–4	2	—	Job Engagement Q14–16	5.22 ± 1.10	1–7	1–1–1	—
Section B—Domain: Workplace policies and culture									
Sup. work culture† Q17–21	2.30 ± 0.81	1–4	0–0–0–1–3	4–11–8–5–0	Health programs Q26A–G	0.44 ± 0.74	0–1	2–2–2–2–2–2–2	75–46–44–54–59–59–49
Management trust† Q22	2.62 ± 0.89	1–4	0	4	Work to non-work conflict Q27	−4.43 ± 1.66	−(1–7)	6	—
Health culture Q23–24	2.12 ± 0.82	1–4	1–1	3–10	Non-work to work conflict Q28	−3.18 ± 1.55	−(1–7)	4	—
Job benefits† Q25A–N	5.58 ± 3.05	0–1	2–2–2–2–2–2–2–2–2–2–2–2–2–2	56–64–60–71–103–42–116–143–24–82–107–87–84–116	Workplace/Schedule flexibility Q29–30	1.62 ± 0.69	1–4	6–2	– 81
Section C—Domain: Workplace physical environment and safety climate									
Workplace safety Q31	2.95 ± 0.62	1–4	4	—	Sexual harassment Q37	−0.04 ± 0.19	−(0–1)	4	—
Safety climate† Q32A–F	2.76 ± 0.91	1–4	2–2–2–2–2–2	15–13–10–11–13–19	Physical violence Q38	−0.21 ± 0.41	−(0–1)	4	—
Work environment satisfaction† Q33A–D	2.56 ± 0.70	1–4	6–6–6–2	– – – 43	Bullying† Q39–40	−0.24 ± 0.34	−(0–1)	5–2	– 9
Discrimination Q34–36	−1.36 ± 0.60	−(1–4)	3–8–6	—					
Section D—Domain: Health status									
Overall health Q41	3.38 ± 0.93	1–5	4	—	Risky drinking Q55	−0.30 ± 0.46	−(0–1)	7	—
Days poor physical health Q42	−4.19 ± 7.85	−(0–30)	9	—	Healthy diet Q56	3.38 ± 1.25	1–6	10	—
Chronic health conditions Q43A–I	−1.37 ± 1.22	−(0–1)	7–6–6–6–6–6–6–6–6	—	Sleep hours Q57	−0.55 ± 0.50	−(0–1)	6	—
Insomnia Q44	−0.30 ± 0.46	−(0–1)	5	—	Sleepy at work Q58	−2.47 ± 1.04	−(1–5)	5	—
Days poor mental health Q45	−5.14 ± 7.98	−(0–30)	10	—	Cognitive functioning limitations† Q59	−1.62 ± 0.76	−(1–4)	3	8
Overall stress Q46A–D	−3.41 ± 1.40	−(1–7)	10–11–10–10	—	Work limitations† Q60	−1.32 ± 0.61	−(1–4)	3	17
Poor mental health Q47–50	−1.55 ± 0.61	−(1–4)	4–6–7–6	—	Productivity Q61A–D	−1.72 ± 0.85	−(1–7)	12–12–13–12	—
Physical activity Q51–52	2.52 ± 1.09	1–7	11–13	—	Work-related injury Q62	−0.04 ± 0.21	−(0–1)	12	—
Tobacco use Q53A–E	−0.53 ± 0.73	−(0–1)	8–9–9–9–8	—	Injury consequence† Q63	−0.14 ± 0.35	−(0–1)	3	147
Alcohol consumption Q54	N/A	0–1	N/A	—					
Section E—Domain: Home, community, and society aspects									
Life satisfaction Q64	3.23 ± 0.66	1–4	6	—	Support outside of work Q67	3.24 ± 0.96	1–4	5	—
Financial insecurity Q65–66	−2.54 ± 0.86	−(1–4)	6–6	—	Activities outside of work Q68A–G	3.99 ± 1.66	0–1	3–3–3–3–3–3–3	8–9–13–12–14–13–12

* For items where the total score is derived from multiple questions, the numerical sequence shown in the “Missing” and “Not Applicable Responses” columns reflects the order of the individual questions.

† Mean scores are calculated taking into account only the applicable responses (for each item is provided the number of applicable responses): Q3 (185), Q5 (194), Q17–Q21 (199), Q22 (196), Q23–Q24 (186), Q25 (187), Q26 (175), Q29–30 (194), Q32 (190), Q33 (196), Q39–Q40 (197), Q59 (189), Q60 (180), Q63 (50), Q68 (189).

In the second domain, which relates to “Workplace policies and culture,” the availability of health programs at work obtained a mean score of 0.44 ± 0.74. This question on the NIOSH WellBQ asks workers to indicate whether their company offers different types of health promotion programs, such as smoking cessation, alcohol and substance or stress management programs, and if company strategies, policies, and infrastructure enable adequate physical activity, healthy eating, and socializing with colleagues. In the best possible scenario (i.e., if the participant responded in the affirmative to all items included in this question), the maximum score would be 7, with an average score of 3.5. Therefore, the mean score obtained in the study would suggest the lack of these tools. Similarly, the results regarding the availability of job benefits offered by the employer showed an average score of 5.58 ± 3.05 (where the possible score range for this question is 0 to 14, with an average of 7). However, these outcomes should be considered with caution because, among the possible answers, the worker could also choose the options “Don’t Know” or “Does not apply” and, indeed (as reported in Section B of Table 2), the number of these not applicable responses was very high, which could also suggest a lack of knowledge of these topics. Another point to consider is that workers would suffer the burden of their job even outside of normal working hours/place because they reported that, rather often, the professional demands interfere with their personal life (−4.43 ± 1.66).

Concerning the “Workplace physical environment and safety climate” and “Health status” domains, the processing of the questionnaires revealed fairly encouraging average scores for the vast majority of the items (Table 2, Sections C and D). In greater detail, the average scores for “Work-related Sexual Harassment,” “Work-related Physical Violence,” and “Work-related Bullying” resulted very low and overall the health conditions of the workers in the study sample showed a relatively low number of days of poor mental and physical health (−5.14 ± 7.98 and −4.19 ± 7.85, respectively) and a very low chronic health conditions mean score (−1.37 ± 1.22, within a range from 0 to 9, and an average of 4.5). Finally, a similar situation was also observed in the fifth domain (Table 2, Section E) whose items investigate the experiences, feelings, and activities of participants outside of work. In this regard, it is noteworthy to point out the meaningful average scores for “Life Satisfaction” and “Support Outside of Work” items (3.23 ± 0.66 and 3.24 ± 0.96, respectively), indicating that this working population is very satisfied with its living conditions and can count on supportive families and social networks.

### Comparison of the WellBQ Mean Scores in Different Areas of the Hospital

One of NIOSH WellBQ’s main prerogatives is the ability to identify the specific health and safety requirements and well-being needs of homogeneous groups of workers within the same facility or workforce, simply by comparing the results obtained in these groups. In this regard, in Table 3, we reported the mean scores for the 68 questionnaire items, categorized by area of work (i.e., surgical, medical, rehabilitation, and outpatient services). Our results showed statistically significant differences in every domain of the NIOSH WellBQ (except the last one), thus providing useful insights into how the available resources should be optimized to improve workers’ well-being in a targeted and effective manner.

**TABLE 3.** Mean Scores and Standard Deviation (SD) of Different Items Grouped According to Healthcare Workers’ Hospital Areas of Expertise

Items	Surgical Divisions (*n* = 70)	Medical Departments (*n* = 50)	Rehabilitation Units (*n* = 44)	Outpatient Services (*n* = 36)	*P* (<0.05)	Items	Surgical Divisions (*n* = 70)	Medical Departments (*n* = 50)	Rehabilitation Units (*n* = 44)	Outpatient Services (*n* = 36)	*P* (<0.05)
Domain: Work evaluation and experience (mean scores ± SD)											
Job satisfaction Q1	3.01 ± 0.69	2.86 ± 0.78	2.86 ± 0.71	3.19 ± 0.66	n.s.	Job autonomy Q8	2.68 ± 0.83	2.58 ± 0.90	2.88 ± 0.76	2.86 ± 0.76	n.s.
Wage satisfaction Q2	2.21 ± 0.70	2.08 ± 0.72	2.04 ± 0.74	2.42 ± 0.84	n.s.	Time paucity/Work overload Q9	−2.90 ± 0.84	−3.46 ± 0.73	−3.09 ± 0.83	−2.69 ± 0.85	n.s.
Benefits satisfaction* Q3	2.11 ± 0.90	2.27 ± 0.98	2.20 ± 0.94	2.56 ± 0.75	n.s.	Meaningful work Q10–11	3.36 ± 0.58	3.63 ± 0.40	3.52 ± 0.52	3.51 ± 0.58	**0.047**
Adv. satisfaction Q4	2.20 ± 0.86	2.35 ± 1.01	2.44 ± 0.88	2.53 ± 0.69	n.s.	Positive affect Q12A-B	4.65 ± 1.19	4.84 ± 1.26	5.03 ± 1.43	4.77 ± 1.37	n.s.
Supervisor support* Q5	3.32 ± 0.67	3.18 ± 0.82	3.05 ± 0.82	3.28 ± 0.95	n.s.	Negative affect Q12E-H	−3.13 ± 1.31	−3.56 ± 1.16	−3.22 ± 1.43	−3.03 ± 1.00	n.s.
Coworker support Q6	3.48 ± 0.65	3.48 ± 0.65	3.5 ± 0.59	3.36 ± 0.96	**0.007**	Work-related fatigue Q13	−4.73 ± 1.25	−5.38 ± 1.35	−4.72 ± 1.46	−4.72 ± 1.20	n.s.
Job security Q7	2.71 ± 0.68	2.36 ± 0.75	2.77 ± 0.84	3.19 ± 0.75	n.s.	Job engagement Q14–16	4.98 ± 1.16	5.38 ± 1.09	5.27 ± 1.09	5.39 ± 0.95	n.s.
Domain: Workplace policies and culture (mean scores ± SD)											
Sup. work culture* Q17–21	3.13 ± 1.31	2.22 ± 0.81	2.34 ± 0.77	2.52 ± 0.88	n.s.	Health programs Q26A–G	0.28 ± 0.67	0.43 ± 0.58	0.43 ± 0.76	0.78 ± 0.93	**0.015**
Management trust* Q22	2.59 ± 0.92	2.50 ± 0.93	2.69 ± 0.81	2.85 ± 0.74	n.s.	Work to non-work conflict Q27	−4.74 ± 1.56	−4.64 ± 1.51	−3.74 ± 1.73	−4.38 ± 1.77	n.s.
Health culture Q23–24	1.80 ± 0.72	2.01 ± 0.76	2.21 ± 0.83	2.51 ± 0.86	n.s.	Non-work to work conflict Q28	−3.12 ± 1.48	−3.04 ± 1.61	−3.16 ± 1.60	−3.16 ± 1.55	n.s.
Job benefits* Q25A-N	5.03 ± 2.67	6.29 ± 2.70	6.20 ± 2.50	6.66 ± 3.23	n.s.	Workplace/Schedule flexibility Q29–30	1.55 ± 0.59	1.74 ± 0.65	1.74 ± 0.77	1.55 ± 0.62	n.s.
Domain: Workplace physical environment and safety climate (mean scores ± SD)											
Workplace safety Q31	2.94 ± 0.52	2.70 ± 0.73	3.02 ± 0.59	3.22 ± 0.54	**0.049**	Sexual harassment Q37	−0.01 ± 0.12	−0.06 ± 0.24	−0.09 ± 0.29	−0.00 ± 0.00	**<0.001**
Safety climate* Q32A–F	2.82 ± 0.78	2.78 ± 0.71	2.87 ± 0.64	3.15 ± 0.70	n.s.	Physical violence Q38	−0.07 ± 0.26	−0.47 ± 0.50	−0.27 ± 0.45	−0.03 ± 0.16	**<0.001**
Work environment satisfaction* Q33A–D	2.73 ± 0.53	2.59 ± 0.63	2.25 ± 0.77	2.73 ± 0.66	n.s.	Bullying* Q39–40	−0.23 ± 0.34	−0.25 ± 0.30	−0.24 ± 0.36	−0.24 ± 0.36	n.s.
Discrimination Q34–36	−1.23 ± 0.45	−1.34 ± 0.59	−1.50 ± 0.73	−1.41 ± 0.71	**0.002**						
Domain: Health status (mean scores ± SD)											
Overall health Q41	3.42 ± 0.80	3.50 ± 0.88	3.45 ± 1.02	3.02 ± 1.02	n.s.	Risky drinking Q55	−0.29 ± 0.46	−0.20 ± 0.40	−0.50 ± 0.50	−0.25 ± 0.44	n.s.
Days poor physical health Q42	−5.09 ± 9.09	−3.00 ± 5.40	−3.07 ± 6.38	−5.38 ± 9.43	**<0.001**	Healthy diet Q56	3.5 ± 1.32	3.24 ± 1.23	3.47 ± 1.32	3.22 ± 1.07	n.s.
Chronic health conditions Q43A–I	−1.23 ± 0.92	−1.18 ± 1.39	−1.59 ± 1.45	−1.50 ± 0.97	**0.001**	Sleep hours Q57	−0.52 ± 0.50	−0.56 ± 0.50	−0.50 ± 0.50	−0.66 ± 0.48	n.s.
Insomnia Q44	−0.23 ± 0.42	−0.26 ± 0.44	−0.35 ± 0.48	−0.44 ± 0.50	n.s.	Sleepy at work Q58	−2.54 ± 1.04	−2.44 ± 1.09	−2.30 ± 1.00	−2.58 ± 1.02	n.s.
Days poor mental health Q45	−6.12 ± 8.78	−4.44 ± 6.87	−4.33 ± 7.66	−5.19 ± 8.37	n.s.	Cognitive functioning limitations* Q59	−1.62 ± 0.75	−1.44 ± 0.68	−1.66 ± 0.78	−1.83 ± 0.84	n.s.
Overall stress Q46A–D	−3.38 ± 1.44	−3.48 ± 1.37	−3.54 ± 1.35	−3.30 ± 1.48	n.s.	Work limitations* Q60	−1.15 ± 0.40	−1.23 ± 0.51	−1.54 ± 0.67	−1.48 ± 0.83	**<0.001**
Poor mental health Q47–50	−1.54 ± 0.54	−1.50 ± 0.53	−1.52 ± 0.67	−1.67 ± 0.74	n.s.	Productivity Q61A–D	−1.71 ± 0.89	−1.56 ± 0.63	−2.00 ± 1.11	−1.66 ± 0.62	**<0.001**
Physical activity Q51–52	2.45 ± 1.98	2.44 ± 1.74	2.20 ± 1.77	2.01 ± 1.67	n.s.	Work-related injury Q62	−0.05 ± 0.21	−0.02 ± 0.14	−0.04 ± 0.21	−0.08 ± 0.28	**<0.001**
Tobacco use Q53A-E	−0.49 ± 0.66	−0.7 ± 0.84	−0.57 ± 0.77	−0.30 ± 0.57	n.s.	Injury consequence* Q63	−0.22 ± 0.42	−0.1 ± 0.31	−0.18 ± 0.40	−0.00 ± 0.00	n.s.
Domain: Home, community, and society aspects (mean scores ± SD)											
Life satisfaction Q64	3.22 ± 0.57	3.22 ± 0.65	3.21 ± 0.77	3.25 ± 0.69	n.s.	Support outside of work Q67	3.26 ± 0.95	3.31 ± 0.94	3.14 ± 0.98	3.25 ± 1.02	n.s.
Financial insecurity Q65–66	−2.56 ± 0.80	−2.63 ± 0.88	−2.39 ± 0.85	−2.55 ± 0.95	n.s.	Activities outside of work Q68A-G	4.01 ± 1.41	4.26 ± 1.40	4.20 ± 1.40	3.91 ± 1.50	n.s.

Significant values are marked with bold type.

* Mean scores are calculated taking into account only the applicable responses (for each item is provided the number of applicable responses). Surgical divisions: Q3 (62), Q5 (68), Q17–Q21 (67), Q22 (70), Q23–Q24 (69), Q25 (64), Q26 (58), Q29–30 (194), Q32 (65), Q33 (67), Q39–40 (69), Q59 (66), Q60 (62), Q63 (21), Q68 (68); medical divisions: Q3 (48), Q5 (50), Q17–Q21 (50), Q22 (50), Q23–Q24 (50), Q25 (48), Q26 (46), Q29–30 (50), Q32 (50), Q33 (50), Q39–40 (49), Q59 (48), Q60 (48), Q63 (10), Q68 (49); rehabilitation units: Q3 (43), Q5 (41), Q17–Q21 (43), Q22 (42), Q23–Q24 (44), Q25 (40), Q26 (40), Q29–30 (35), Q32 (41), Q33 (43), Q39–40 (44), Q59 (43), Q60 (42), Q63 (11), Q68 (41); outpatient services: Q3 (32), Q5 (36), Q17–Q21 (36), Q22 (34), Q23–Q24 (36), Q25 (35), Q26 (31), Q29–30 (35), Q32 (34), Q33 (36), Q39–40 (36), Q59 (36), Q60 (33), Q63 (11), Q68 (34).

n.s., not significant.

For example, although workers in all areas of work are fairly satisfied with the support they receive from colleagues, it is worth noting that there is a statistically significant difference in the average score for this item (*P* = 0.007). Similarly, in the same domain, all workers are well aware of the significance and crucial role of their work, but this perception, despite remaining largely positive (average score of 3.36 ± 0.58), is less recognized and felt among employees working in the surgical divisions (*P* = 0.047). In the “Workplace policies and culture” domain, we observed only one statistically significant difference related to the “Availability of Health Programs at Work” index (*P* = 0.015), where workers in the medical departments and rehabilitation units had a mean score in line with that of the entire working population (0.43 ± 0.58, 0.43 ± 0.76, and 0.44 ± 0.74, respectively). Unfortunately, considering the high number of nonapplicable responses and the inability to conduct secondary statistical analyses (by stratifying the study population according to the main sociodemographic characteristics), we are currently unable to give a precise meaning to these differences or speculate on their possible reasons. Some interesting findings have been observed in the “Discrimination” scale and the “Work-related Sexual Harassment” and “Work-related Physical Violence” items of the third domain, where we obtained statistically significant differences (*P* = 0.002, *P* < 0.001, and *P* < 0.001, respectively). In this regard, it is worth noting that almost half of the medical department workers and nearly one-third of the rehabilitation unit workers reported an episode of work-related physical violence in the last 12 months. Finally, in the “Health Status” domain, statistically significant differences were detected in the “Days of Poor Physical Health” and “Work-related Injury” items, “Chronic Health Conditions” index, and “Productivity” scale.

## DISCUSSION

The findings of this study confirm the NIOSH WellBQ’s ability to capture the multiple nuances and complex interactions between work and nonwork factors and contexts that might affect the well-being of healthcare workers. These findings are consistent with the original validation study, which conducted a psychometric analysis of the NIOSH WellBQ and demonstrated its applicability across diverse sectors, including healthcare, social assistance, education, retail, and professional services.^24^ Several features underscore the tool’s psychometric robustness. Among those, the questionnaire items were derived from established instruments designed to measure relevant constructs, supporting face validity. The items encompassed a wide range of recognized indicators of worker well-being, contributing to content validity. Factor analyses revealed well-defined constructs with strong model fit and internal consistency. Finally, the observed relationships among measures aligned with theoretical expectations and prior research, confirming concurrent, convergent, and discriminant validity. Additionally, the comparison across domains is justified by the structure of the NIOSH WellBQ itself, which is designed to assess multiple facets of worker well-being. Previous literature supports the use of domain-level analysis to understand workplace health comprehensively.^25^ Our interpretation of relative domain is based on standardized scoring and distribution patterns observed in our sample, which align with the instrument’s intended use. However, the NIOSH guidance on interpretation of questionnaire data points out that the available information is insufficient for developing algorithms to create summary scores aimed to characterize worker well-being, nor does consent absolute or clinical judgments on this parameter,^25^ taking into consideration the different scoring ranges of the 68 questions in the NIOSH WellBQ. It is noteworthy that only in seven items the average scores obtained are below the respective mean scoring ranges (Table 2).

In greater detail, concerning the time paucity/work overload (Q9 of Table 2), findings highlighted that most of the participants agreed, to some extent, that they never have enough time to get everything done on their job, and this figure is consistent with (and may even explain) the data observed in the first domain, which is related to the work-related fatigue (Q13 of Table 2). These factors are perceived as unfavorable conditions that warrant attention, along with satisfaction related to wages, benefits, and opportunities for advancement. However, these data are not unexpected, given that both work-related overload and fatigue are common conditions among workers in the healthcare sector.^26,27^ Moreover, similar concerns among healthcare workers were highlighted in another study utilizing the NIOSH WellBQ, further supporting the tool’s validity and reliability in assessing dimensions of worker well-being.^20^ These situations experienced by healthcare workers are influenced by a complex combination of work-related (night and shift work, demanding and safety-critical tasks, long working hours), individual (age, job experience, chronic health conditions, sleep quality and duration), and psychosocial factors (poor job control and autonomy, lack of coworkers or supervisors support, perceived stress), which consequently require a thorough analysis of the determinants and a multidisciplinary approach to potential solutions.^27–30^ Overall, considering the results obtained in the questions related to some of the aforementioned factors (colleague/supervisor/social support, job engagement, meaningful work, chronic health conditions, insomnia, days of poor physical and/or mental health, overall stress, and job/life satisfaction), our data would seem to suggest that priority should be given to the organizational and procedural aspects of work. This may mean to implement measures aimed at ensuring an adequate staffing level, regular and well-planned shifts and schedules, a limitation on long working hours, and a periodic and timely training/coaching. Furthermore, the possibility of defining and implementing administrative or managerial initiatives to improve worker satisfaction on important issues such as wages, advancement, and benefits should also be explored.

In fact, regarding the last topic, the low overall satisfaction reported by participants is consistent with the low mean score concerning the employer’s provision of job benefits. From a TWH approach perspective, in which worker well-being is pursued through programs and practices that combine the prevention and protection of professional risk factors with health promotion (taking into account all occupational, work-related, and individual determinants and their possible interplays and synergies that can negatively impact workers’ health), these topics are of key importance in ensuring a flourishing working environment.^8,31^ Therefore, in this regard, there is a clear need for greater commitment to defining and implementing company policies and strategies that should increase benefits for employees, as well as initiatives and programs promoting mental and physical health, which (also in consideration of the results obtained in the “Health Status” domain) should be particularly targeted on combating sedentary lifestyles, teaching stress management, and support sleep hygiene. However, it should be emphasized that correctly interpreting these results requires acknowledging the high frequency of nonapplicable responses (“Don’t Know” or “Does not apply” choices) in both the 14-item index of “Availability of job benefits” and the 7-item index of “Availability of health programs.” For example, in the first of these subdomain constructs, the 35.5% and 21% of the respondents provided a nonapplicable response at the items “Paid maternity leave” and “Paid sick leave,” respectively. These figures are even more surprising when bearing in mind that several benefits indicated in the question (such as those just mentioned) are actually guaranteed workers’ rights in Italy. Then, although a greater availability of job benefits and workplace health promotion initiatives and/or programs is desirable, based on these observations, it would be necessary to investigate these two areas in further detail. In fact, the data would also suggest the possibility that the low average scores on these topics may be due to workers’ lack of knowledge. In that case, the employer’s efforts should be oriented toward a more extensive and effective outreach and information campaign to make workers aware of the benefits, tools, and opportunities that are already available to them.

It is also important to point out that, according to the World Health Organization (WHO), health is “a state of complete physical, mental and social well-being and not merely the absence of disease or infirmity.”^32^ In Occupational Medicine (also taking into account what the WHO stated in 1986 on the occasion of the first International Conference on Health Promotion),^33^ this concept should extend to viewing a worker as healthy when they experience psychophysical and social well-being, enabling full use of their physical, emotional, and mental capacities and not merely the absence of occupational illness or injury. However, in order to achieve this key goal, there is a need to use a participatory approach that encourages workers to actively participate in improving health and safety conditions at work, as well as their overall well-being. This bottom-up participatory model, that is, a fundamental principle of the TWH model, should ensure worker’s involvement in identifying safety and health issues, contributing to the design of improvement measures and strategies, and participating in the various aspects of their implementation and subsequent evaluation.^8,34^ Using a bottom-up approach to involve workers in workplace safety, health, and well-being has many advantages.^35^ Firstly, it enables issues considered critical by workers (and by specific groups of workers within the same workforce) to be prioritized, while also helping to identify obstacles to implement potential corrective measures, thus ultimately improving their long-term sustainability.^8,34,35^ In this context, the NIOSH WellBQ is a truly valuable resource, as it ensures workers’ direct involvement in all the aforementioned areas and, at the same time, allows for the definition and proper implementation of improvement measures and tools tailored to their real needs. However, it should be noted that often, especially in highly structured, complex, and specialized work facilities, where the production cycle is based on the sequential and coordinated execution of multiple job tasks and work activities (performed by different types of workers), the needs and requirements of these categories of workers do not necessarily coincide. For this reason, we considered it important to compare results within various worker subpopulations to identify specific and category-targeted aspects of worker well-being in need of special attention.

In this regard, our findings suggest that it would be beneficial to improve support among colleagues, particularly among outpatient service workers, and this goal could be achieved by promoting greater socialization in the workplace through an increase in common spaces or activity hubs.^32,36,37^ Similarly, a winning strategy could be to promote company initiatives that facilitate teamwork, even outside the normal working context and schedule. Furthermore, this type of initiative has the advantage of being able to pursue multiple goals at once. For example, based on the results obtained, the possibility of organizing sports tournaments in which workers could participate in teams organized according to their working area could be explored. This kind of initiative would have the potential to strengthen bonds between colleagues, thereby facilitating mutual support, but at the same time, it would also meet the need to promote a healthy lifestyle, particularly by encouraging physical activity. Moreover, associating these tournaments with reward mechanisms (e.g., company benefits such as bonus leave or gym membership discounts) could improve employee satisfaction and encourage widespread participation. The analysis of mean scores for the “Discrimination” scale and the “Work-related Sexual Harassment” and “Work-related Physical Violence” items is another example of how comparing WellBQ results across different groups of workers can suggest specific and tailored intervention measures, because the statistical elaboration revealed significant differences among the various work areas. Although the very low response rate to the optional questions prevents us from formulating explanatory hypotheses regarding the causes of these differences, it can be speculated that they stem from the heterogeneous nature of the healthcare services provided, as medical and rehabilitation wards require close, continuous, and particularly complex interactions with patients (and their families) in challenging and critical conditions. Therefore, although these issues did not appear to be particularly significant across the overall workforce, the evaluation of data, subdivided by hospital areas of expertise, suggests that any corrective or improvement measures should be prioritized for workers engaged in medical departments and rehabilitation units. Additionally, these findings align with a previous study that used the NIOSH WellBQ and identified concerns related to workplace physical violence, work-related sexual harassment, and bullying among clinicians,^20^ as well as with earlier published investigations,^38–40^ reinforcing the validity of the tool and the need for comprehensive and targeted interventions.

In recent years, the issue of workplace violence in the healthcare system (with verbal assaults being the most frequent type, mainly in psychiatric wards and emergency departments) has become a real social scourge that can have extremely serious consequences for their psychological and physical health and well-being.^41–44^ Therefore, in light of these considerations, it is not surprising that these data were discussed at length with and among the workers and aroused the interest of the employer and of the OSH system at the meetings held at the end of the study to present the results. In this regard, it should be emphasized that the employer deemed it appropriate to take immediate action to identify and apply solutions to counteract the problem of workplace violence, and consequently, in this perspective, it is currently testing the dead man’s device (model GH5200, Teltonika, Kaunas, Lithuania) that, based on the study data, has been distributed primarily to medical and rehabilitation workers. This device is capable of detecting falls or immobility of the worker and promptly sending an alarm signal that activates the rescue and emergency system. Its usefulness is therefore twofold, as it allows for immediate assistance to a worker in the event of physical assault but also in the event of a fall or immobility resulting from a health problem (that is particularly helpful in particular working circumstances such as the night shift).

**FIGURE 1.**
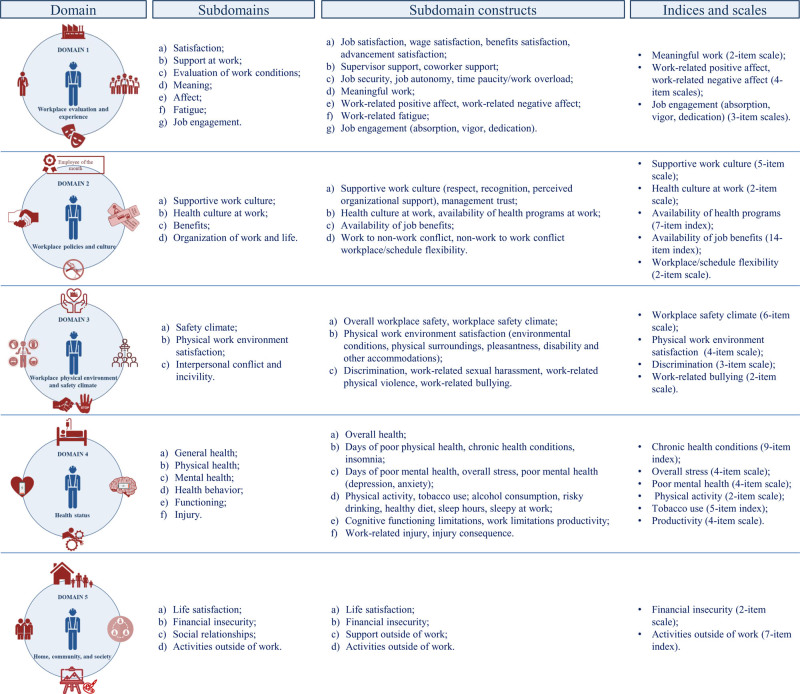
Domains, subdomains, subdomain constructs, indices, and scales of the WellBQ. The work evaluation and experience domain includes 4 scales and 10 single items. The workplace policies and culture domain includes 3 scales, 2 indices, and 3 single items. The workplace physical environment and safety climate domain includes 4 scales and 3 single items. The health status domain includes 4 scales, 2 indices, and 13 single items. The home, community, and society domain includes 1 scale, 1 index, and 2 single items.

Although this study provides valuable insights, contributing to the existing body of research in this field, it is important to acknowledge some limitations that should be stressed in order to inform methodologically adequate investigation in the future. The NIOSH WellBQ includes five main domains organized into subdomains and domain constructs (Fig. 1).^22–25^ Given the recent development of the questionnaire, it is evident that its administration in different professional settings is still quite limited, and thus, the lack of sufficient and reliable data prevents the development of algorithms to define summary scores for categorizing workers’ well-being. Moreover, it should be emphasized that NIOSH’s guidelines on the proper use and interpretation of the instrument clearly stated that its scores are not useful in establish absolute or clinical judgments about workers’ well-being, and at the same time, they do not identify thresholds or cutoff levels that might inform about the need for action to improve it.^25^ From this perspective, our methodology for evaluating concerning responses relied on mean item scores and group comparisons. Although this approach was deemed reasonable by consensus among researchers, it may not represent the most accurate method for categorizing levels of concern. Nevertheless, a baseline assessment using the NIOSH WellBQ in a company can be extremely useful for various OSH professionals because, by providing detailed information on the numerous determinants that can negatively affect the well-being of the workforce, it allows the designing and implementation of prevention, protection, and improvement interventions and strategies focused on those aspects that have been found to be most critical. Furthermore, in this context, the periodic administration of the tool (especially following the implementation of a specific intervention) enables the monitoring of changes in well-being, provides reliable quantitative indicators of the intervention’s effectiveness, and uncovers causal relationships among the factors influencing clinicians’ well-being. Finally, it must be noted that not all homogeneous groups of workers within the same facility or workforce have the same health, safety, and welfare needs.

Our ability to statistically process the results was significantly impacted by our inability to stratify the study population based on key sociodemographic characteristics. Consequently, with the exception of the working areas, it was not possible to compare the results of the questionnaire between specific groups of workers based on variables such as gender, age, education, marital status, and annual income, and this represents a considerable limitation, especially with regard to the possibility of identifying critical issues specific to each variable and thus designing tailored improvement measures. In this regard, taking into account the need to guarantee workers’ anonymity, these analyses are essentially based on the information participants provide by answering the final section of the questionnaire, called “Optional Items.” In fact, these items concern key work-related information and sociodemographic characteristics, and although they are derived from common occupational health survey models used for research or surveillance applications, their data can be useful also for practical workplace applications. In our experience, the vast majority of workers left this section blank, which could be due to several reasons or a combination of them. For example, it is possible that its placement at the end of the questionnaire may discourage participants from completing this part, in which, moreover, being defined as “Optional Items,” could be perceived by workers as less important and therefore facultative to fill out. In this regard, placing this section at the beginning of the questionnaire might encourage participants to complete it more thoroughly and thoughtfully, although it should be noted that, from a psychometric perspective, positioning it at the end of the tool is generally considered the more appropriate approach for collecting these data. Finally, given the cross-sectional design of this study and the entirely voluntary nature of participant recruitment, it is important to emphasize that our data are not able to establish cause-effect relationships, and the possibility of self-selection or confirmation biases cannot be excluded.

## CONCLUSIONS

The connection between workplace health, safety, and well-being has become a growing area of research, receiving an increasing attention from OSH researchers and professionals in last years. This has led to a broader definition of worker well-being as an integrative concept reflecting quality of life, shaped by health and work-related environmental, organizational, and psychosocial factors.^23,45^ In this framework, preventing occupational diseases and accidents (traditionally achieved through risk assessment and management process) remains essential, but is no longer sufficient. A broader, interdisciplinary approach is needed to address the wide range of factors (occupational, work-related, social, and individual) that influence worker health, safety, and well-being. Indeed, given the numerous and varied determinants that can influence well-being, as well as the potential beneficial effects that can result from the definition of multidisciplinary strategies and policies aimed at improving it, it is reasonable to assume that this topic will continue to be an extremely timely and interesting area of research in the future. However, the feasibility of devising measures and initiatives to improve this complex parameter must necessarily be based on the availability of robust and reliable data that should be studied, compared, and analyzed by the scientific community. This possibility, in turn, depends on the use of validated and effective tools capable of adequately measuring well-being and, at the same time, accounting for its many facets and complexities.

In this context, our findings showed that the NIOSH WellBQ is a suitable tool for achieving the aforementioned objective. What we find most impressive is the questionnaire’s ability to capture the various nuances that can influence well-being, and thus, performing a fully comprehensive and multidisciplinary assessment, it allows for the specific identification of the most critical areas where intervention is required. Indeed, in our experience, the information provided by this tool on individual items, scales, or indices has allowed us to identify the weakest and most sensitive issues that should be addressed as a priority in order to improve the well-being both of all healthcare workers and of specific groups of them. In this regard, it should be noted that, from a health, safety, and well-being perspective, the ability to rationalize and optimize improvement interventions in line with the specific needs of workers and targeting them at those who need them most is a fundamental advantage, especially in those work contexts (such as small- and medium-sized enterprises) where both human and economic resources are limited.

Obviously, given the current scarcity of information in the literature, further studies are needed on this and other working populations to confirm the NIOSH WellBQ’s validity, compare results, and share possible improvement policies, programs, and practices. In particular, it would be desirable to conduct longitudinal studies involving the readministration of the questionnaire to assess the effectiveness of interventions applied in the workplace that have been defined based on an initial administration of the questionnaire itself.
